# Deep learning‐based analysis of *EGFR* mutation prevalence in lung adenocarcinoma H&E whole slide images

**DOI:** 10.1002/2056-4538.70004

**Published:** 2024-10-02

**Authors:** Jun Hyeong Park, June Hyuck Lim, Seonhwa Kim, Chul‐Ho Kim, Jeong‐Seok Choi, Jun Hyeok Lim, Lucia Kim, Jae Won Chang, Dongil Park, Myung‐won Lee, Sup Kim, Il‐Seok Park, Seung Hoon Han, Eun Shin, Jin Roh, Jaesung Heo

**Affiliations:** ^1^ Department of Radiation Oncology Ajou University School of Medicine Suwon Republic of Korea; ^2^ Department of Biomedical Sciences Graduate School of Ajou University Suwon Republic of Korea; ^3^ Department of Otolaryngology Ajou University School of Medicine Suwon Republic of Korea; ^4^ Department of Otorhinolaryngology‐Head and Neck Surgery Inha University College of Medicine Incheon Republic of Korea; ^5^ Division of Pulmonology, Department of Internal Medicine Inha University College of Medicine Incheon Republic of Korea; ^6^ Department of Pathology Inha University College of Medicine Incheon Republic of Korea; ^7^ Department of Otolaryngology‐Head and Neck Surgery Chungnam National University Hospital Daejeon Republic of Korea; ^8^ Division of Pulmonary, Allergy and Critical Care Medicine, Critical Care Medicine, Department of Internal Medicine Chungnam National University Hospital Daejeon Republic of Korea; ^9^ Division of Hematology and Oncology, Department of Internal Medicine Chungnam National University Hospital Daejeon Republic of Korea; ^10^ Department of Radiation Oncology Chungnam National University Hospital Daejeon Republic of Korea; ^11^ Department of Otorhinolaryngology‐Head and Neck Surgery Hallym University Dontan Sacred Heart Hospital, Hallym University College of Medicine Hwaseong Republic of Korea; ^12^ Department of Pathology, Dongtan Sacred Heart Hospital Hallym University College of Medicine Hwaseong Republic of Korea; ^13^ Department of Pathology Ajou University School of Medicine Suwon Republic of Korea

**Keywords:** *EGFR*, whole‐slide image analysis, deep learning in histopathology, multiple‐instance learning

## Abstract

*EGFR* mutations are a major prognostic factor in lung adenocarcinoma. However, current detection methods require sufficient samples and are costly. Deep learning is promising for mutation prediction in histopathological image analysis but has limitations in that it does not sufficiently reflect tumor heterogeneity and lacks interpretability. In this study, we developed a deep learning model to predict the presence of *EGFR* mutations by analyzing histopathological patterns in whole slide images (WSIs). We also introduced the *EGFR* mutation prevalence (EMP) score, which quantifies *EGFR* prevalence in WSIs based on patch‐level predictions, and evaluated its interpretability and utility. Our model estimates the probability of EGFR prevalence in each patch by partitioning the WSI based on multiple‐instance learning and predicts the presence of *EGFR* mutations at the slide level. We utilized a patch‐masking scheduler training strategy to enable the model to learn various histopathological patterns of EGFR. This study included 868 WSI samples from lung adenocarcinoma patients collected from three medical institutions: Hallym University Medical Center, Inha University Hospital, and Chungnam National University Hospital. For the test dataset, 197 WSIs were collected from Ajou University Medical Center to evaluate the presence of *EGFR* mutations. Our model demonstrated prediction performance with an area under the receiver operating characteristic curve of 0.7680 (0.7607–0.7720) and an area under the precision‐recall curve of 0.8391 (0.8326–0.8430). The EMP score showed Spearman correlation coefficients of 0.4705 (*p* = 0.0087) for p.L858R and 0.5918 (*p* = 0.0037) for exon 19 deletions in 64 samples subjected to next‐generation sequencing analysis. Additionally, high EMP scores were associated with papillary and acinar patterns (*p* = 0.0038 and *p* = 0.0255, respectively), whereas low EMP scores were associated with solid patterns (*p* = 0.0001). These results validate the reliability of our model and suggest that it can provide crucial information for rapid screening and treatment plans.

## Introduction

Epidermal growth factor receptor (*EGFR*) is a crucial gene for cellular regeneration and survival, and it plays key roles in tissue repair and maintaining the cellular microenvironment [1, 2, 3, 4]. However, mutations in the *EGFR* gene are linked to various cancers, including non‐small cell lung cancer (NSCLC), gallbladder cancer, and glioblastoma [5, 6, 7]. Therefore, EGFR is an important target for cancer treatment. In particular, the development and clinical application of EGFR tyrosine kinase inhibitors (EGFR‐TKIs) have significantly improved the survival and clinical outcomes of NSCLC patients with *EGFR* mutations. Consequently, EGFR‐TKIs are recommended as the standard first‐line treatment for patients with these mutations [8, 9]. It is crucial to rapidly identify patients with mutations to administer the appropriate treatment in a timely manner. Although next‐generation sequencing (NGS) has enabled relatively fast mutational genomic diagnostics [10], challenges such as difficulty in obtaining sufficient samples and high costs persist [11].

In contrast, machine‐learning‐based whole slide image (WSI) analysis has shown promise in various medical fields [12, 13, 14, 15, 16, 17, 18] and has demonstrated satisfactory performance in predicting mutational genomics [16, 19, 20, 21]. Predicting mutational genomics using WSI is time‐ and cost‐effective and, because it utilizes previously collected samples, it has the potential to be an effective alternative to mutational genomic testing.

In machine learning, a multiple‐instance learning (MIL) approach is employed for WSI analysis [22]. MIL is a type of weakly supervised learning method that trains patch‐level prediction models using slide‐level labels. This enables the learning of the spatial heterogeneity of tumors and the prediction of slide‐level probabilities by aggregating patch‐level predictions. MIL is known to be effective for WSI analysis [23, 24, 25, 26] and has been utilized to successfully predict the mutational genomics of various cancer types. For instance, MIL‐based approaches have been applied to predict *EGFR* mutations in lung adenocarcinoma [20, 21], genetic alterations in gastric cancer [27], and mutation status in breast carcinoma [28]. Moreover, a hierarchical deep MIL model to predict gene mutations in bladder cancer has been reported [19]. These studies demonstrate the potential of MIL in capturing the spatial heterogeneity of tumors and predicting clinically relevant mutations from histopathology images across various cancer types.

MIL can learn patch‐level probabilities through weakly supervised learning using only slide‐level labels. We hypothesized that, by utilizing this feature, we could obtain the probability of *EGFR* mutation in the entire tumor region and calculate the *EGFR* prevalence in WSI. To validate our hypothesis, we collected histopathological patterns (lepidic, acinar, solid, papillary, and micropapillary) from pathology reports and analyzed the allele frequencies obtained through DNA mutation testing, indirectly measuring the significance of the predicted region. The proposed method effectively addresses the limitations of previous studies [20, 21], which provided only binary predictions of *EGFR* mutation status, by predicting the prevalence of *EGFR* mutations.

## Materials and methods

### Data collection

We retrospectively selected data from patients with histopathologically confirmed lung adenocarcinoma who underwent *EGFR* mutation testing (PCR or NGS) at four large independent institutions: Hallym University Medical Center (HUMC) (*n* = 145), Inha University Hospital (INHA) (*n* = 129), Chungnam National University Hospital (CNUH) (*n* = 397), and Ajou University Medical Center (AJMC) (*n* = 197) from 2012 to 2022. We collected one WSI per patient, totaling 868 WSIs. Detailed information on the cohort and the relationship between clinical data and *EGFR* mutations is presented in Table 1, and detailed information on the WSIs is shown in Table 2.

**Table 1:** Details of cohort

Clinical characteristics		AJMC (*n* = 197)			HUMC (*n* = 145)			CNUH (*n* = 397)			INHA (*n* = 129)		
		*EGFR* wild‐type (*n* = 69)	*EGFR* mutant (*n* = 128)	*p*	*EGFR* wild‐type (*n* = 80)	*EGFR* mutant (*n* = 65)	*p*	*EGFR* wild‐type (*n* = 235)	*EGFR* mutant *(n* = 162)	*p*	*EGFR* wild‐type *(n* = 67)	*EGFR* mutant (*n* = 62)	*p*
Gender	Female	26	85	0.0001	29	41	0.0023	85	104	0.0001	24	45	0.0001
	Male	43	43		51	24		150	58		43	17	
Smoking status	Y	52	107	0.2272	53	53	0.0605	97	92	0.0032	32	51	0.0001
	N	17	21		27	12		128	70		35	11	
Family history	Y	66	126	0.477	74	59	0.736	209	128	0.0101	49	46	0.9493
	N	3	2		5	6		26	34		18	16	
Age (mean)		66.09 ± 9.94	64.10 ± 9.78	0.0941	65.15 ± 11.07	66.34 ± 12.51	0.3934	67.19 ± 9.34	66.37 ± 9.433	0.1505	63.17 ± 10.94	62.98 ± 12.01	0.4727
Height (mean)		162.69 ± 8.09	158.78 ± 9.15	0.0007	161.40 ± 8.51	159.21 ± 7.33	0.0404	162.01 ± 8.73	158.65 ± 8.28	0.0001	163.17 ± 7.07	157.89 ± 7.85	0.0003
Weight (mean)		62.85 ± 10.68	59.37 ± 10.93	0.0195	62.84 ± 10.67	59.58 ± 9.51	0.0334	64.50 ± 10.32	60.77 ± 10.24	0.0001	65.04 ± 11.64	62.23 ± 9.39	0.1813
Stage	I	44	101	0.0832	46	41	0.2866	189	133	0.3522	41	46	0.2664
	II	11	14		7	7		26	18		8	8	
	III	11	8		12	12		10	2		16	7	
	IV	3	5		15	5		10	9		2	1	

The clinicopathological characteristics of cohorts of lung adenocarcinoma patients collected from four institutions, AJMC, HUMC, CNUH, and INHA, are summarized according to *EGFR* mutations. The statistical significance of differences between groups was evaluated by the chi‐square test or *t*‐test.

**Table 2:** Details of WSIs

		AJMC	HUMC	CNUH	INHA
Number of patients		197	145	397	129
Number of WSIs		197	145	397	129
Scanning magnification	×20	197	126	69	129
	×40	0	19	328	0
Microns per pixel		0.5025 ± 0.0003	0.4695 ± 0.0847	0.2479 ± 0.0027	0.5000 ± 0.0
Width		52,138 ± 7,377	58,413 ± 23,900	82,714 ± 7,562	46,961 ± 8,096
Height		40,922 ± 6,179	42,932 ± 16,835	114,027 ± 19,933	56,232 ± 10,464
Machine	Aperio	197	145	397	129
Number of patches (1,024 × 1,024 pixel, ×20 magnification)	Tumor	140,857	77,805	213,844	79,628
	Non‐tumor	39,981	25,176	140,585	50,438

This table describes the details of the whole slide images (WSIs).

To further validate the developed model, we collected histopathological pattern information from pathology reports (*n* = 197) and allele frequency data from NGS results (*n* = 68) in the AJMC cohort. The predominant histopathological pattern was defined as a pattern (lepidic, acinar, solid, papillary, or micropapillary) occupying more than 50% of the WSI, as described by the pathologist in the pathology report. The AJMC cohort was chosen as the test dataset because it provided additional information for evaluating the predictive performance of the model from multiple perspectives. The Institutional Review Board (IRB) of Ajou University Hospital approved this study (AJOUIRB‐MDB‐2022‐249). Furthermore, the requirement for informed consent from all participants was waived by the IRB because of the retrospective nature of this study. All methods were performed in accordance with the Declaration of Helsinki.

### Preprocessing

The collected WSIs were scanned using Aperio equipment. All WSIs were standardized to ×20 magnification using the Lanczos filter [29] to ensure uniform cell and tissue sizes and then divided into fixed‐size (1,024 × 1,024 pixels) patch images for use as input images for MIL.

To develop and evaluate the tumor region segmentation model, the tumor regions were annotated by experienced pathologists specializing in lung cancer diagnosis and pathological examination. Pathologists with over 3 years of clinical experience meticulously labeled the boundaries of the cancerous tissue by using the polygon tool in the open‐source software QuPath [30].

The patches were divided into non‐tumor and tumor regions, and background areas that were not tissue regions were excluded using Otsu's adaptive thresholding method [31] as per prior studies [32]. Variations in the tissue staining and scanning processes between medical centers can occur. Methods such as stain normalization [33] and Macenko normalization [34] have been proposed to address this, but we proposed RandStainNA [35], which showed more diverse staining enhancement. The method described above was applied to the patch image for learning. We used ViT‐B/14, a variant of the vision transformer (ViT), to extract features from the divided patch images. This model was pretrained using the DINO algorithm on the ImageNet dataset [36]. DINO is an unsupervised learning technique that uses self‐distillation to train models and is commonly used with ViT models. It has also been successfully applied to various medical image analysis tasks. For instance, Wessels *et al* [37] employed a self‐supervised ViT pretrained with DINO to predict survival from histopathology images in renal cell carcinoma, thereby demonstrating its effectiveness in capturing prognostic information. Similarly, Li *et al* [38] utilized a DINO‐pretrained ViT for the weakly supervised histopathological image analysis of primary brain tumors, showcasing its ability to learn discriminative features from limited labeled data. These studies highlight the potential of DINO‐pretrained ViT models in extracting meaningful representations from complex medical images.

**Figure 1:**
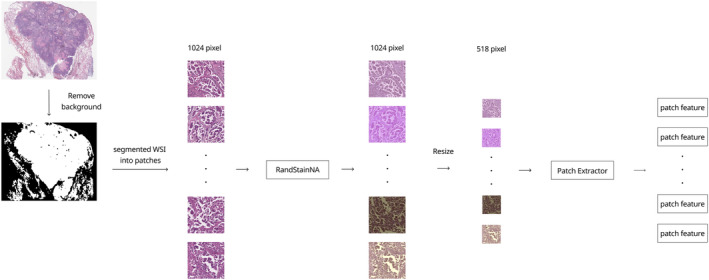
Preprocessing of WSIs. Mimetic diagram illustrating the preprocessing process of WSI data. Series of steps: extraction of patch images from the original WSI, RandStainNA, and feature extraction. The ViT‐B/14 DINO model was used for feature extraction.

In this study, the pretrained ViT‐B/14 model with all frozen parameters was used to extract high‐dimensional features from the patch images. The input image size was down‐sampled to 518 × 518 pixels to fit the ViT model. The extracted features were used for subsequent analyses, including the prediction of *EGFR* mutations. The detailed workflow from WSI extraction to patch image feature extraction is illustrated in Figure 1.

### Tumor region segmentation

To analyze *EGFR* mutations in the WSIs, we first performed tumor region segmentation. The model was trained using the WSSS4LUAD dataset [39]. WSSS4LUAD is a publicly available dataset specifically designed for the weakly supervised semantic segmentation of lung adenocarcinoma histopathology images. It consists of WSIs annotated with patch‐level labels for tumor and non‐tumor regions and is therefore appropriate for tumor region segmentation. We classified the patch images into tumor and non‐tumor regions using a binary classification model. The model used for the classification was ViT‐B/14.

### MIL with patch‐masking strategy

**Figure 2:**
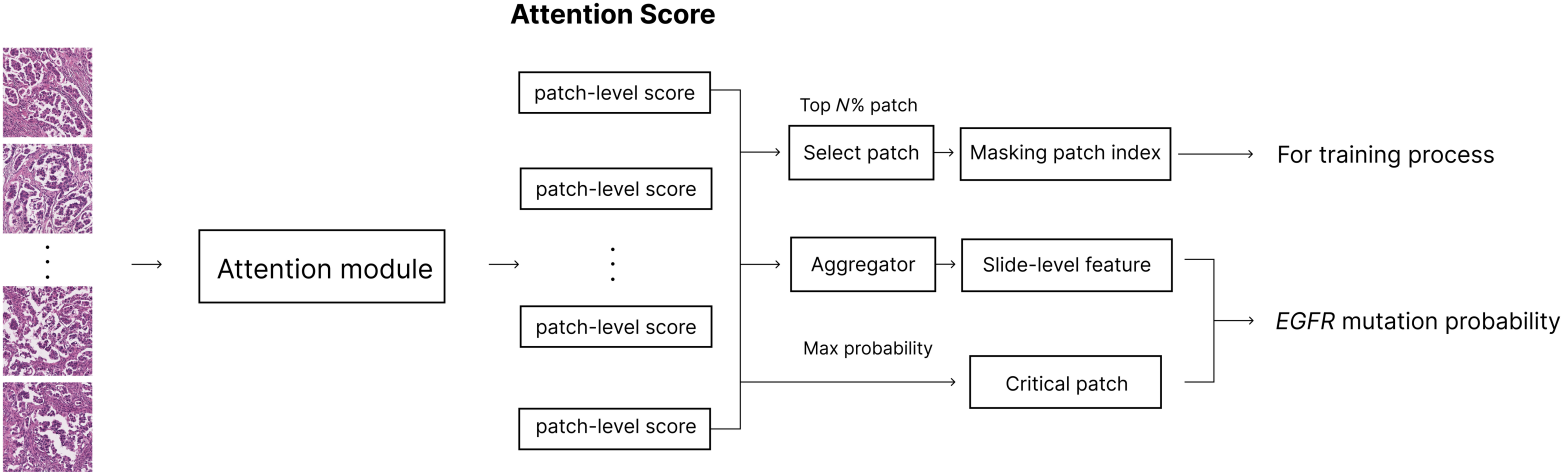
MIL architecture. Structure of dual‐stream multiple‐instance learning (DSMIL) model used for predicting *EGFR* mutations in this study. DSMIL learns features of the patch with the highest probability of *EGFR* mutation and aggregates slide‐level features based on the attention score. During the training phase, the teacher model performs masking based on the predicted score.

For predicting *EGFR* mutations, we used the MIL model, specifically the dual‐stream MIL (DSMIL) model. The DSMIL consists of a two‐stream architecture. The first stream identifies the critical instance that is expected to be most related to *EGFR* mutations among the patches. The second stream calculates the attention score by measuring the distance between each instance and the critical instance, thereby evaluating the importance of each instance. This approach is robust even when the proportion of positive patches is low, thereby addressing the data imbalance issues [22]. Considering the need to accurately detect a small number of patches related to EGFR mutations, the DSMIL model was selected for this study. The structure of the DSMIL used in this study is shown in Figure 2.

**Figure 3:**
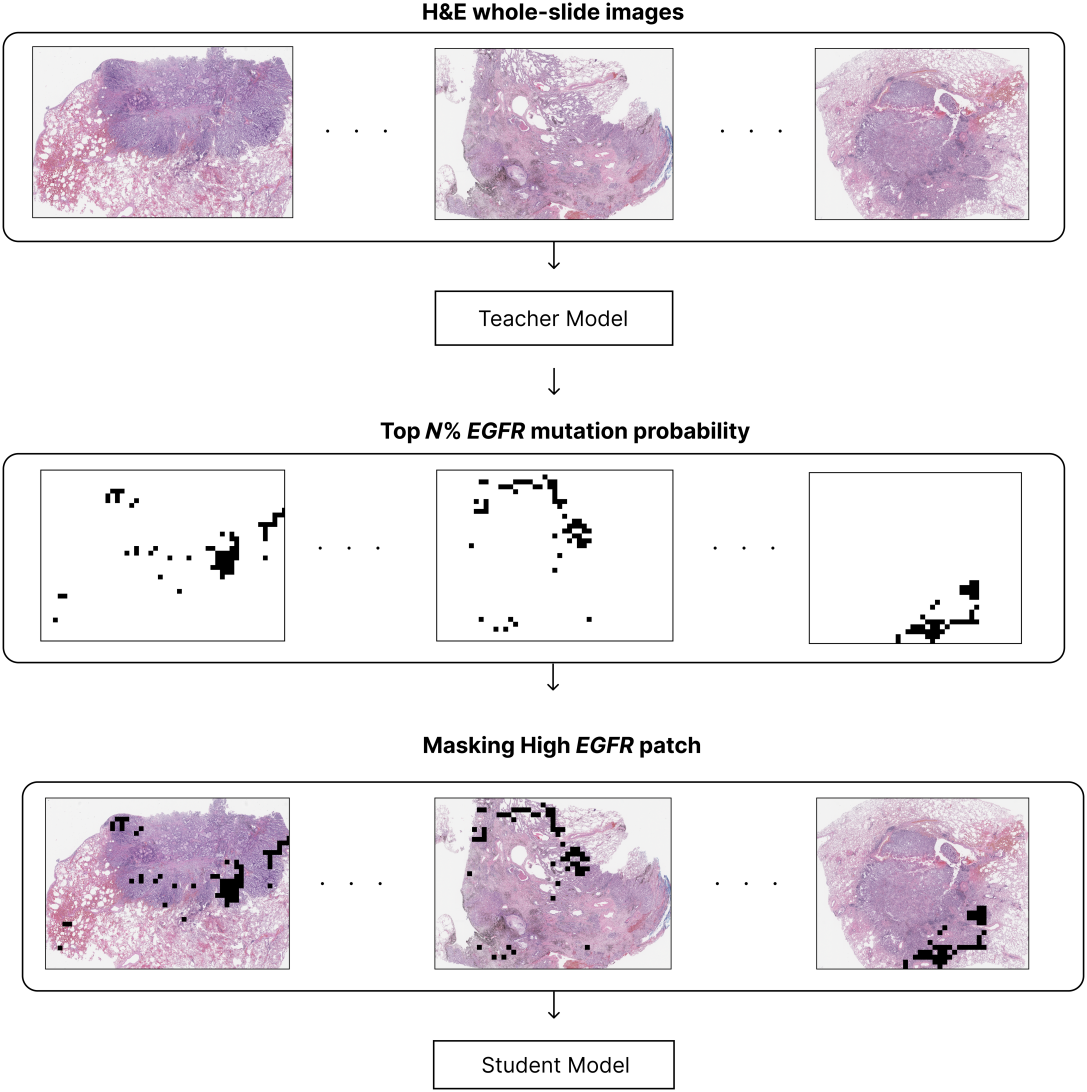
Attention‐based masking. Process of masking the top *N*% of patches with high *EGFR* mutation rate. By masking these patches, the model can focus on learning various patterns, including the easy‐to‐distinguish features of *EGFR* mutation.

We also adopted masked hard‐instance mining [40] to train the model on various histopathological patterns exhibiting *EGFR* mutations. This training strategy uses two models: teacher and student. The teacher model first calculates the patch‐level attention score, which indicates the relevance of each patch for the *EGFR* mutation. The student model receives masked patches as input and learns various histopathological patterns. Figure 3 illustrates the process of masking the top *N*% of patches based on the attention scores, focusing the training on the more challenging and diverse histopathological patterns that are less indicative of *EGFR* mutations.

**Figure 4:**
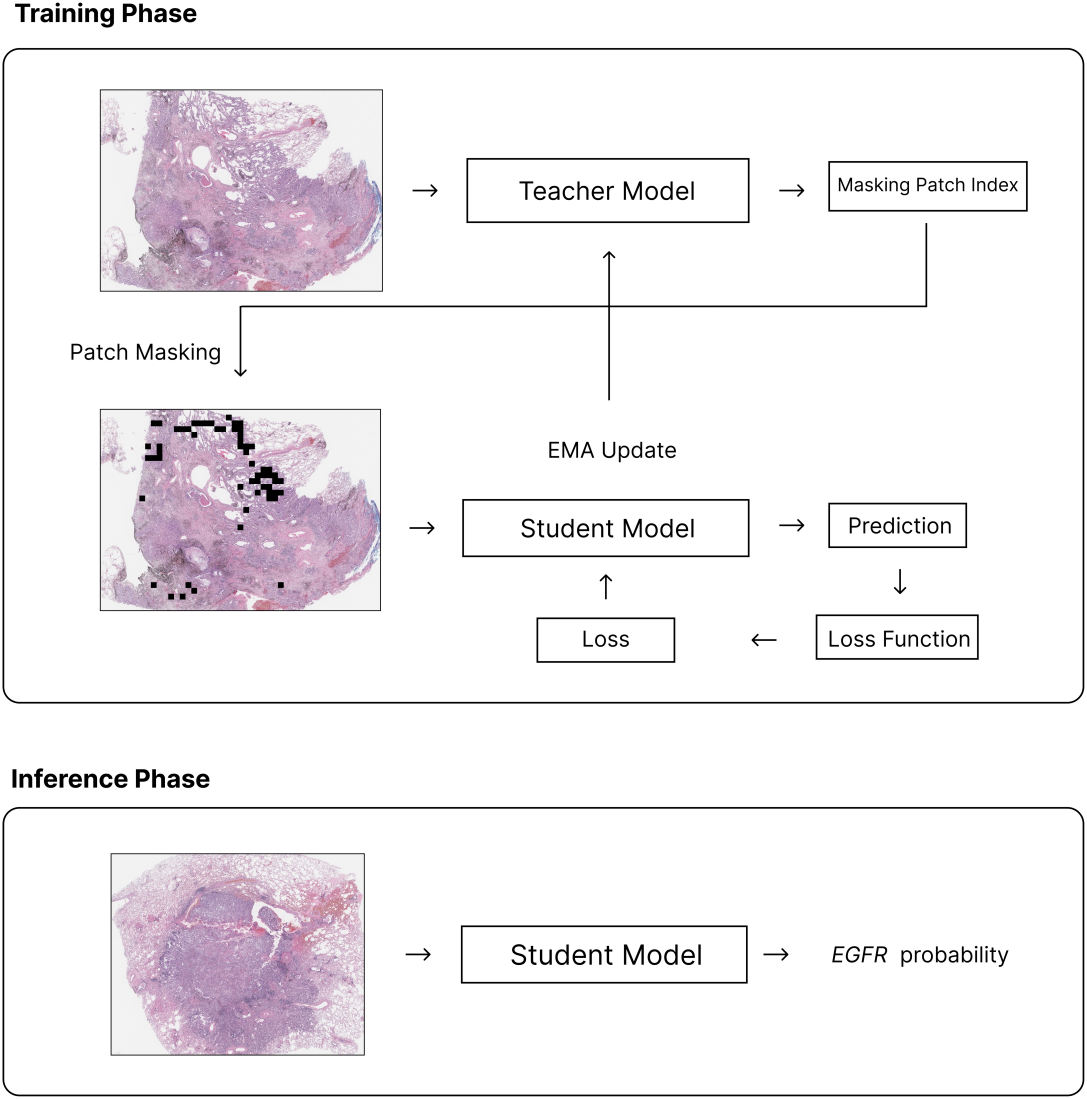
Training and inference process. Process of learning and reasoning using MIL and patch‐masking strategies. A teacher model is utilized for learning various patterns during the training phase and is not involved in the reasoning process.

However, the fixed‐ratio patch‐masking method can lead to the loss of common and easily classified histopathological patterns of *EGFR* mutations during the early stages of training. Therefore, we introduced a patch‐masking scheduler that dynamically adjusts the masking ratio as training progresses. This allows the model to learn the characteristics of tissues with high *EGFR* mutation presence early in the training and gradually increases the masking ratio to learn various tissue patterns as the training continues. This process enables the model to initially learn the easier histopathological features and then the more complex *EGFR* patterns as the training progresses. The teacher model was used only during the training process and not during the inference process. The training and inference processes using the proposed model are shown in Figure 4.

### Artificial intelligence‐based *EGFR* mutation prevalence score in tumor areas

In the process of learning the presence of *EGFR* mutations at the slide level, the MIL model learns the prediction probabilities at the patch level. We proposed an artificial intelligence (AI)‐based *EGFR* mutation prevalence (EMP) score that quantifies the prevalence of *EGFR* mutation in the tumor region of WSIs. This score is the ratio of the sum of the *EGFR* mutation probability values predicted by the model at the patch level to the total number of patches in the tumor region, as shown in the formula below:

$$\mathrm{EMP}\;\text{score}=\frac{\sum_{i=1}^{m}M_{i}}{n}.$$

where n represents the total number of patches and $M_{i}$ represents the score of the ith patch among the m patches where the presence of *EGFR* mutation is predicted. By dividing the sum of the prediction probabilities $\sum_{i=1}^{m}M_{i}$ by the total number of patches n, we obtained the EMP score, which represents the prevalence of tumor regions containing *EGFR* mutations in the WSIs.

## Results

### Tumor region classification performance

The tumor region classification model, trained using the WSSS4LUAD dataset, was evaluated using expert annotations in our cohort. In the training data (CNUH, INHA, and HUMC, *n* = 868), the model achieved an area under the receiver operating characteristic curve (AUROC) of 0.9048 (0.8826–0.9210) and an F1 score of 0.7628 (0.7482–0.7871). In the test data (AJMC, *n* = 197), the model achieved an AUROC of 0.8922 (0.8714–0.9224) and an F1 score of 0.7632 (0.7486–0.7822) (Table 3). For EMP, following common practice in medical image analysis [41, 42], only patch images with a tumor prediction probability above 0.5 were used. If no patches in a WSI had a probability above 0.5, we sequentially applied lower thresholds, and no WSIs had tumor patches with a probability below 0.3.

**Table 3:** Performance of tumor classification

Metric	Tumor classification	
	Training cohort (HUMC, INHA, CNUH)	Test cohort (AJMC)
AUROC	0.9048 (0.8826–0.9210)	0.8922 (0.8714–0.9224)
F1 score	0.7628 (0.7482–0.7871)	0.7632 (0.7486–0.7822)
Accuracy	0.8321 (0.8126–0.8488)	0.8251 (0.8018–0.8432)

A performance table of tumor/non‐tumor region classification models learned using the WSSS4LUAD dataset. Indicators such as AUROC, F1 score, and accuracy were used in the training datasets (HUMC, INHA, and CNUH) and the test/verification datasets (AJMC).

### Performance of deep learning model in predicting *EGFR* mutation

In this study, we proposed a patch‐masking scheduler learning strategy that allows the model to learn easy features related to *EGFR* presence early in training and more difficult features as the training progresses. Our model achieved an AUROC of 0.7680 (0.7607–0.7720) and an area under the precision‐recall curve of 0.8391 (0.8326–0.8430) in the AJMC cohort (*n* = 197), outperforming traditional MIL methods (Table 4).

**Table 4:** Performance of MIL models in *EGFR* mutation prediction

	Ours	MHIM	DSMIL	ABMIL
AUROC	0.7680 (0.7607–0.7720)	0.7441 (0.7381–0.7508)	0.7210 (0.7170–0.7302)	0.7021 (0.6982–0.7094)
AUPRC	0.8391 (0.8326–0.8430)	0.8098 (0.7983–0.8124)	0.7904 (0.7862–0.8022)	0.7622 (0.7584–0.7712)

Table comparing the performance of multiple‐instance learning (MIL) models developed for *EGFR* mutation prediction. AUROC and AUPRC values of the proposed model (Ours) and existing methods (MHIM, DSMIL, and ABMIL) are presented.

AUPRC, area under the precision‐recall curve.

### Evaluating EMP score in tumor areas

We quantitatively measured the AI‐based EMP score, which represents the prevalence of *EGFR* in the WSIs based on patch‐level predictions. We evaluated the significance of the EMP score by comparing it with the variant allele frequency (VAF) of *EGFR* mutations using NGS and predominant histopathological patterns.

### Correlation between EMP score and allele frequency

Spearman's correlation analysis was performed to compare the relationship between the EMP score and VAF in tumor regions (*n* = 64). The two variables showed a correlation of 0.3708 (*p* = 0.00679), indicating that higher EMP in tumor tissue corresponded to a higher frequency of *EGFR* mutations in DNA, despite not showing a strong correlation.

**Figure 5:**
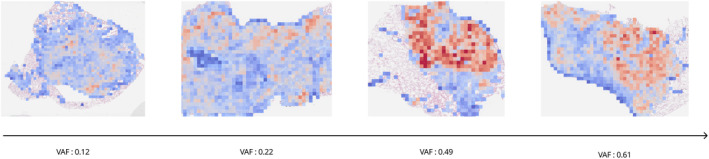
*EGFR* prevalence heatmaps (EMP scores) and VAFs. Heat maps showing the probability of *EGFR* mutation predicted by the AI model for each WSI. They visually represent that higher VAF scores correspond to more areas predicted to be *EGFR* mutation positive.

We also compared the results based on clinically significant *EGFR* mutation subtypes, exon 19 deletion (exon 19 del) and p.L858R, known for their distinct histopathological patterns [43, 44] and treatment responses [45]. Using fewer larger patches (1,024 × 1,024 pixel patches from ×20 magnification), exon 19 del (*n* = 22) showed a correlation of 0.59184 (*p* < 0.00371), whereas exon 21 p.L858R (*n* = 30) did not show any correlation (0.1704, *p* = 0.3689). Conversely, using numerous smaller patches (512 × 512 pixel patches from ×20 magnification), p.L858R showed a correlation of 0.4705 (*p* = 0.00877), but exon 19 del did not (0.0412, *p* = 0.8553) (Table 5). Figure 5 visually presents the EMP score as a heatmap based on patch‐level prediction probabilities, showing that higher VAF scores tend to correspond to larger regions of *EGFR* mutation presence.

**Table 5:** Spearman correlation between VAF and EMP score

	512 x 512 pixel patches		1024 x 1024 pixel patches	
	Coefficient	*p* value	Coefficient	*p* value
Total	0.3038	0.0285	0.3708	0.0067
p.L858R	0.4705	0.0087	0.1704	0.3689
exon 19 del	0.0412	0.8553	0.5918	0.0037

Correlation between the variant allele frequency (VAF) and the EMP score of *EGFR* mutations by Spearman correlation analysis. The differences according to patch size (512 x 512 / 1024 x 1024 pixels) and *EGFR* mutation subtype are shown.

### EMP score and histopathological pattern analysis

Table 6 presents the results of the chi‐square test analysis of the histopathological patterns of the WSIs and the presence of *EGFR* mutations. *EGFR*‐positive samples showed a significant relationship with the lepidic pattern (*p* = 0.0479), and *EGFR* wild‐type samples showed a significant relationship with the solid pattern (*p* = 0.0047), which is consistent with previous studies [46]. We divided the samples into low and high groups based on the median EMP score and analyzed the predominant histopathological patterns (Table 7). A solid pattern was observed significantly more in the group with low EMP scores (*p* = 0.0001). Papillary (*p* = 0.0038) and acinar (*p* = 0.0255) patterns were observed significantly more in the group with high EMP scores.

**Table 6:** Clinical analysis based on predominant growth pattern and *EGFR* mutation

Predominant growth pattern		*EGFR* wild‐type (*n* = 69)	*EGFR* mutation (*n* = 128)	*p* value
Lepidic	N	68	115	0.0479
	Y	1	13	
Solid	N	53	118	0.0047
	Y	16	10	
Acinar	N	28	36	0.1049
	Y	41	92	
Papillary	N	64	120	1
	Y	5	8	
Micropapillary	N	66	125	0.7291
	Y	3	3	

Results of distribution difference analysis by chi‐square test of predominant histopathological growth patterns (lepidic, solid, acinar, papillary, and micropapillary) according to the presence or absence of *EGFR* mutations.

**Table 7:** Predominant growth pattern and EMP score

Predominant growth pattern		EMP low	EMP high	*p* value
Lepidic	N	92	91	0.7967
	Y	7	7	
Solid	N	76	95	0.0001
	Y	23	3	
Acinar	N	40	24	0.0255
	Y	59	74	
Papillary	N	98	86	0.0038
	Y	1	12	
Micropapillary	Y	96	95	0.6876
	N	3	3	

Comparison results of the high/low group distribution of the *EGFR* mutation prevalence (EMP) score according to each histopathological growth pattern. The EMP score is an index that quantifies the prevalence of *EGFR* mutations in the WSI predicted by the AI model.

## Discussion

In this study, we developed an MIL‐based deep learning model to analyze the prevalence of *EGFR* mutations in WSIs of patients with lung adenocarcinoma. Several studies have confirmed that specific histopathological patterns appear in tumors that contain *EGFR* mutations. In 2009, Ninomiya *et al* reported that micropapillary patterns were associated with *EGFR* mutations [47]. In 2014, Kadota *et al* reported that the lepidic‐predominant group was associated with *EGFR* mutations (*p* < 0.011), whereas papillary‐ and solid‐predominant tumors were associated with wild‐type *EGFR* [46]. More recently, in 2021, Saito *et al* reported that the pure micropapillary nests group was associated with *EGFR* mutations, whereas the small solid nests group was associated with wild‐type *EGFR* [48]. These examples suggest the possibility of predicting *EGFR* mutations through the histopathological pattern analysis of WSIs. Furthermore, previous reports [49, 50] on the close association between histopathological patterns of *EGFR* mutations and the therapeutic effects of *EGFR*‐TKIs further highlight the importance of histopathological pattern studies using WSIs.

However, tumor heterogeneity is a major challenge when predicting mutations using WSIs. Not all tumor cells contain the same genetic alterations [51], and *EGFR* mutations are only observed in a subset of tumor cells [52]. Existing MIL models are limited in that they focus on learning patches with strong features based on attention scores [22] and fail to learn diverse histopathological patterns [46, 47, 48, 49, 50] arising from tumor heterogeneity. To address this problem, we introduced a masking strategy based on patch probabilities [40] during the learning process. We advanced the existing patch‐masking learning technique using a patch‐masking scheduler strategy that dynamically adjusts the masking ratio according to the learning progress rather than using a fixed masking ratio. Through this, our model focuses on learning the features of patches with a high probability of *EGFR* mutation presence in the early stages of learning and gradually includes patches with lower probabilities as learning progresses. This strategy enables the model to comprehensively learn diverse and heterogeneous histopathological features associated with *EGFR* mutations.

In MIL, extracting diverse features at the patch level is crucial, and convolutional neural network (CNN) models are primarily used [53, 54, 55, 56]. However, CNNs have the limitation of primarily focusing on local features within a patch, while ignoring the global context and relative positional information of the cells [57, 58, 59]. Recently, ViTs [60] have gained attention as alternatives to CNNs. ViTs extract features by reflecting cell‐to‐cell interactions through a self‐attention mechanism and position encoding [58, 61]. Furthermore, ViTs can generate high‐level representations without additional fine‐tuning through unsupervised learning techniques such as DINO [36].

We demonstrate the effectiveness of our methodology by utilizing a large‐scale dataset collected from a multi‐institutional cohort. Prior to predicting *EGFR* mutations, we developed a model to classify tumor and non‐tumor regions in WSIs. Through this process, the *EGFR* mutation prediction model considers only the patch images predicted as tumors for *EGFR* mutation prediction. This two‐stage approach minimizes the influence of non‐tumor tissues on WSI analysis and allows the model to focus on learning the characteristics of the tumor region. The developed model showed superior performance to existing MIL‐based models (Table 4). This suggests that the patch‐masking scheduler strategy is effective for our dataset. Future studies with larger cohorts, including more lepidic cases, would be needed to further investigate the relationship between the lepidic subtype and EMP score predicted by the AI model.

When the allele frequency of *EGFR* mutations is low, other pathways are more likely to be involved in tumor pathogenesis. Therefore, at the treatment strategy development stage, it is necessary to test for alterations in other druggable targets in addition to *EGFR*. Previous studies have focused primarily on predicting the presence of *EGFR* mutations. However, our study predicted the EMP score, that is, the degree of EMP, using an AI model. By utilizing this, medical professionals can predesign the scope of genetic testing for patients, preventing unnecessary medical expenses. With future technological advancements, personalized treatment strategies can be offered to patients.

Our study has some limitations. First, we trained the model using only Korean lung cancer tissue data, which does not reflect the characteristics of diverse races and regions. Because the histopathological patterns of lung cancer may vary according to race and region, validation using data from other countries and races is necessary. However, our study demonstrated some degree of generalizability using data collected from four large institutions.

Second, we confirmed that the deep learning model identified *EGFR* mutation regions differently depending on the *EGFR* subtype (exon 19 del, p.L858R) and size and number of patches. This suggests that each *EGFR* subtype has distinct histopathological characteristics that may manifest differently depending on patch size. To elucidate the clinical significance of these findings, future studies will focus on classifying *EGFR* mutation subtypes based on the histopathological features differentially identified by the deep learning model at various patch size levels. Third, we measured the prevalence ratio of *EGFR* mutations in the entire tumor region and compared it with VAF and histopathological patterns. However, considering the intratumoral genetic heterogeneity, it is important to examine the distribution of mutations at the single‐cell level. A comparison of the genetic profiles measured at the individual cell level by single‐cell sequencing with the regions of *EGFR* mutation presence predicted by our model could further strengthen the utility of the model. However, the application of current single‐cell sequencing technologies to large‐scale analyses is difficult owing to cost and technical limitations. Nevertheless, we anticipate that our study, which utilizes deep learning to explore tumor heterogeneity at the single‐cell level, will serve as a foundation for future research in this area.

In conclusion, the proposed method has great potential for mutation prediction from WSIs using DL. The prediction of *EGFR* mutation status demonstrates the possibility of its use as a rapid and primary screening tool in clinical settings, particularly in scenarios where tissue samples for molecular testing are unavailable. Additionally, the method used to measure the prevalence of *EGFR* mutations in the entire tissue has the potential to establish prognosis and treatment plans for patients with *EGFR* mutant tumors. The learning methods and models we propose are effective in considering various aspects of genomic analysis in WSIs and can be utilized in various WSI analyses.

## Author contributions statement

JHP, JH and JR conceived and designed the analysis. JHP, JHyL, SHK, C‐HK, J‐SC, JHeL, LK, JWC, DP, SK, I‐SP, SHH, ES and JR collected the data. JHP and JH contributed to data or analysis tools. JHP performed the analysis. JHP and JH wrote the paper. JH and JR edited the manuscript.

## Data Availability

The datasets generated and/or analyzed in this study are available from the corresponding author upon reasonable request.
